# Contributions to the morphology and phylogeny of the newly discovered bat tick species, *Ixodes ariadnae* in comparison with *I. vespertilionis* and *I. simplex*

**DOI:** 10.1186/s13071-015-0665-0

**Published:** 2015-01-24

**Authors:** Sándor Hornok, Jenő Kontschán, Agustín Estrada-Peña, Isabel G Fernández de Mera, Snežana Tomanović, José de la Fuente

**Affiliations:** Department of Parasitology and Zoology, Faculty of Veterinary Science, Szent István University, Budapest, Hungary; Plant Protection Institute, Centre for Agricultural Research, Hungarian Academy of Sciences, Budapest, Hungary; Department of Zoology and Animal Ecology, Szent István University, Gödöllő, Hungary; Department of Animal Pathology, University of Zaragoza, Zaragoza, Spain; SaBio. Instituto de Investigación en Recursos Cinegéticos IREC, CSIC-UCLM-JCCM, Ciudad Real, Spain; Department for Microbiology and Parasitology, Laboratory for Medical Entomology, Institute for Medical Research, University of Belgrade, Belgrade, Serbia; Department of Veterinary Pathobiology, Center for Veterinary Health Sciences, Oklahoma State University, Stillwater, USA

**Keywords:** *Ixodes*, Tick, Bat, *12S rDNA*, *16S rDNA*, Subolesin

## Abstract

**Background:**

Recently a new hard tick species, *Ixodes ariadnae* has been discovered, adding to the two known ixodid tick species (*I. vespertilionis* and *I. simplex*) of bats in Europe.

**Findings:**

Scanning electron microscopic comparison of adult females of these species shows morphological differences concerning the palps, the scutum, the Haller’s organ, the coxae, as well as the arrangement and fine structure of setae. Molecular analysis of 10 geographically different isolates revealed 90-95% sequence homology in the 12S and 16S rDNA genes of bat tick species. Based on 12S rDNA sequences, genotypes of *I. ariadnae* clustered closest to *I. simplex*, whereas according to their 16S rDNA gene they were closest to *I. vespertilionis*. The subolesin gene of *I. ariadnae* had only 91% sequence homology with that of *I. ricinus*, and is the longest known among hard tick species.

**Conclusions:**

The present study illustrates the morphology and clarifies the phylogenetic relationships of the three known bat tick species that occur in Europe. According to its subolesin gene *I. ariadnae* may have a long evolutionary history.

## Findings

### Background

In Europe and in the majority of the Old World, for more than a century two ixodid ticks were known to be highly specialized to bats, i.e. the bat tick (*Ixodes simplex*) and the long-legged bat tick (*Ixodes vespertilionis*) [1]. Recently, however, a new tick species has been discovered to parasitize chiropterans, hitherto reported only from Hungary [2]. The significance of bat ticks is increased by the fact that bats frequently live close to (or in) human dwellings, and at least *I. vespertilionis* may feed on humans [3] and has been reported to be a potential vector of zoonotic bartonellae [4].

In the description of *I. ariadnae* [2] it was pointed out that it shares features with both *I. vespertilionis* (e.g. its relatively large size and long legs) and *I. simplex* (e.g. its short, broad palps). However, it was beyond the scope of the latter study to illustrate these similarities and differences with appropriate scanning micrographs of all three bat tick species. In addition, although the most important gene that is used for barcoding (species identification) among ticks [5], i.e. the cytochrome oxidase subunit I (COI) gene of *I. ariadnae* was shown to differ considerably from those of *I. vespertilionis* and *I. simplex* [2], examination of the taxonomic relationship of closely related species should optimally include analysis of multiple genes. Therefore it was decided to extend the previous results by a simultaneous morphological comparison of all three bat tick species, together with the molecular analysis of three further (12S/16S rDNA and subolesin) genes.

### Methods

The morphology of two adult females of each of the three bat tick species was compared: *Ixodes vespertilionis* and *I. ariadnae* were collected in Hungary; *I. simplex* was collected in Serbia. For scanning electron microscopy (performed as in [2]) females were chosen, because (a) larvae and nymphs of all three species were not available, and (b) in the adult form females are parasitic: males either do not suck blood (*I. vespertilionis*) or are unknown (*I. ariadnae*) [2].

Total nucleic acid (TNA) was extracted as described [6]. Ten TNA extracts from isolates of different geographical origin were used for molecular studies: four of *I. vespertilionis*, four of *I. ariadnae*, and two of *I. simplex* [2]. From these samples it was attempted to amplify fragments of the 16S and 12S rDNA genes, as well as the subolesin gene as reported [7-9]. PCR products were resin purified (Wizard, Promega) and cloned into the pGEM-T vector (Promega, Madison, WI, USA) for sequencing both strands (Secugen S.L., Madrid, Spain). Obtained sequences were submitted to the GenBank (accession numbers: KM455956-65 for 12S genotypes A-J, respectively; KM455966-70 for 16S genotypes A, B, E, F, I, respectively, and KM455971 for subolesin gene). Phylogenetic analyses were conducted according to the Tamura-Nei model [10] and Maximum Composite Likelihood method by using MEGA version 5.2 [11].

### Ethical approval

Authorization for bat capture was provided by the National Inspectorate for Environment [2].

### Results and discussion

#### Scanning electron microscopy of females

**Figure 1:**
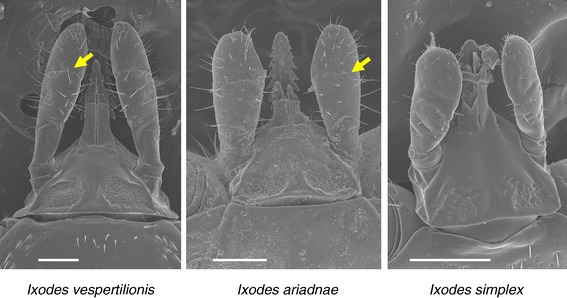
**Dorsal view of the gnathosoma of the three bat tick species.** Arrows indicate separation of II.-III. palpal segments (bars: 200 μm).

Concerning the gnathosoma of the three bat tick species, the palps of *I. vespertilionis* are the longest and narrowest (Figure 1). On the other hand, *I. ariadnae* and *I. simplex* have short, broad palps; in the latter species with indistinct separation of segments II-III and only short hair (Figure 1). The teeth on the hypostome of *I. vespertilionis* point backward. This species has sharp posterolateral flange on the basis capituli, whereas in *I. ariadnae* it is less produced. The two areae porosae of *I. vespertilionis* and *I. ariadnae* are circumscribed, with densely situated, small pits; unlike those of *I. simplex* with scattered pits (Figure 1).

**Figure 2:**
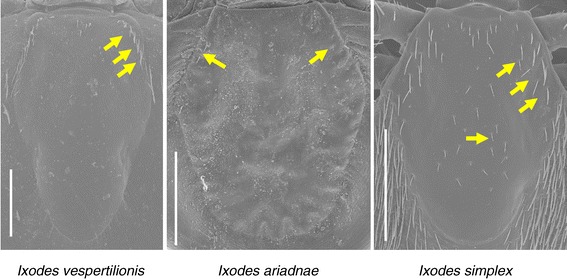
**Dorsal view of the scutum of the three bat tick species.** Arrows indicate hair-covering (bars: 500 μm).

**Figure 3:**
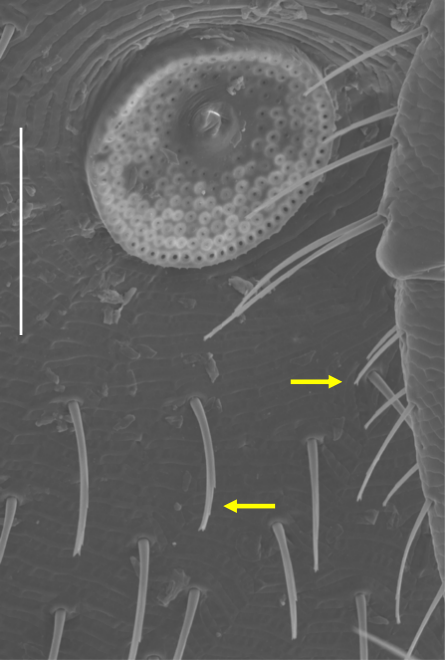
**Pinnate, hair-like setae of** ***Ixodes simplex*** **around the peritreme (bar: 200 μm).**

**Figure 4:**
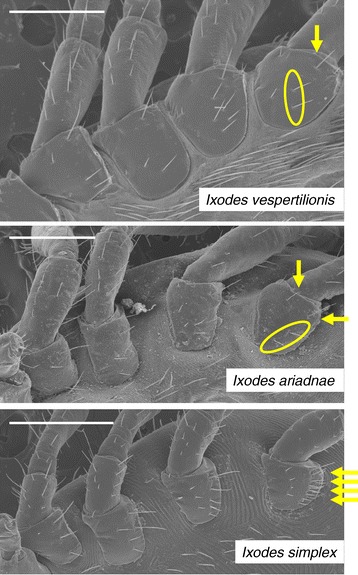
**Ventrolateral aspect of the idiosoma of the three bat tick species.** Characteristic arrangements of setae on coxae IV are encircled or indicated by arrows (bars: 500 μm).

**Figure 5:**
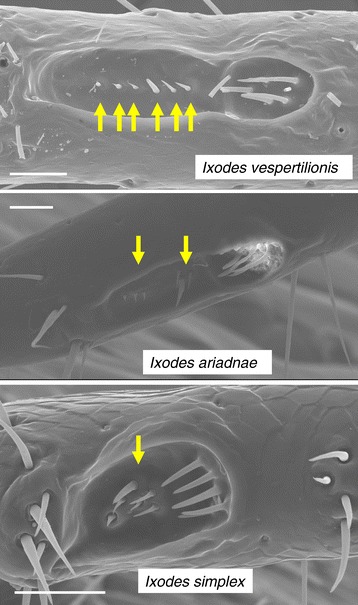
**Haller’s organ of the three bat tick species.** Arrows indicate arrangement of anterior pit sensillae (bars: 50 μm).

On the idiosoma the scutum is the narrowest in *I. vespertilionis*, and broadest (especially posteriorly) in *I. ariadnae* (Figure 2); in the latter species (as contrasted to the other two) with only scarce hair-covering anterolaterally. In case of *I. simplex* middle scutal hairs are also present (Figure 2). Pinnate, hair-like setae were only noted on *I. simplex* (Figure 3). The coxae of *I. ariadnae* are posteroexternally convex (Figure 4), and the arrangement of coxal setae is different in all the three species (Figure 4). Consistently with the long legs of both *I. vespertilionis* and *I. ariadnae*, their Haller’s organ is also elongated (Figure 5). The arrangement of anterior pit sensillae is linear in *I. vespertilionis*, whereas focal in two groups in the case of *I. ariadnae* and in one group in the case of *I. simplex* (Figure 5).

#### Molecular analyses

Recently it has been suggested that for molecular identification of tick species sequencing of the COI gene should be the first method of choice, and analysis of 12S and 16S rDNA genes can be performed as complementary tests [5,7,8]. Prior to this study there was only one 12S (and no 16S) sequence of *I. vespertilionis* in GenBank (U95909), and for *I. simplex* only other genes have been published [12,13]. Therefore sequences obtained in the present study compensate for this lack of information on genetic markers in case of bat tick species.

**Figure 6:**
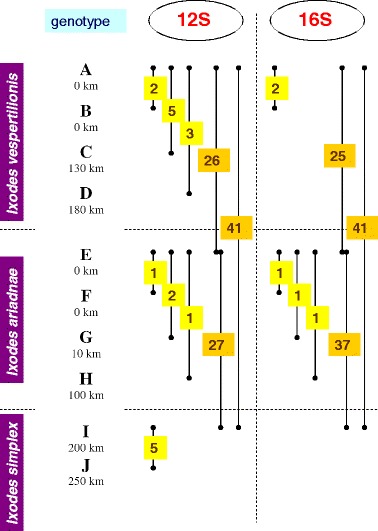
**Sequence heterogeneity of the 12S and 16S rDNA genes of bat tick isolates.** Genotypes marked with capital letter are shown next to the relevant tick species. Below each genotype the distance from the most important collection site of *I. ariadnae* is indicated. Data are separated by dashed line according to genotypes (12S or 16S) and tick species. The number of nucleotides differing between pairs of genotypes is marked with yellow, whereas inter-specific differences with orange background. Sequencing was successful for both genes in case of all 10 isolates, but on the diagram only differences are shown.

Because it is more likely that DNA samples of different geographical origin will show sequence divergence, as demonstrated for both the COI [2] and 12S/16S rDNA genes of ticks [14], tick specimens that were used for molecular genetic comparison in this study were collected at different locations (Figure 6).

**Figure 7:**
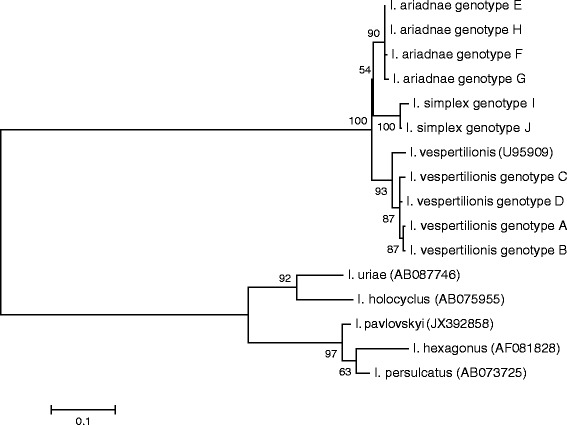
**Phylogenetic comparison of 12S rDNA sequences of bat tick genotypes identified in the present study and related sequences from the GenBank.** Branch lengths correlate to the number of substitutions inferred according to the scale shown.

**Figure 8:**
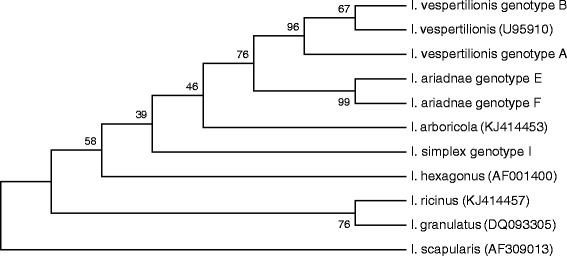
**Phylogenetic comparison of 16S rDNA sequences of bat tick genotypes identified in the present study and related sequences from the GenBank.**

Molecular analyses revealed 90-95% sequence homology in the 12S and 16S rDNA genes of bat tick species (Figure 6). In particular, according to the 12S rDNA gene, the difference between *I. ariadnae* and *I. vespertilionis* is of similar magnitude to that between *I. ariadnae* and *I. simplex* (26 vs. 27 nucleotide differences: 94% vs. 93% sequence homology, respectively: Figure 6). However, in the 16S rDNA gene of its isolates, *I. ariadnae* appears to be more closely related to *I. vespertilionis*, than to *I. simplex* (25 vs. 37 nucleotide differences: 95% vs. 92% sequence homology, respectively: Figure 6). In their 12S and 16S genes *I. vespertilionis* and *I. simplex* differed with 41 nucleotides (90% and 91% homology, respectively). With bootstrap analyses, isolates/genotypes of the same bat tick species always clustered together, but separately from isolates of other bat tick species. Based on its 12S rDNA sequences *I. ariadnae* clustered closest to *I. simplex* (Figure 7), whereas according to the 16S rDNA gene it neighbored *I. vespertilionis* (Figure 8).

Concerning differences between genotypes of the same bat tick species, *I. ariadnae* had the lowest sequence heterogeneity in its 12S gene (with up to two nucleotide differences), as contrasted to the other two bat tick species (with up to five nucleotide differences) (Figure 6). In line with this, the COI sequences were also highly conserved between isolates of *I. ariadnae* [2]. The 12S sequences of geographically distant specimens were always different, unlike those of the 16S rDNA gene (Figure 6).

**Figure 9:**
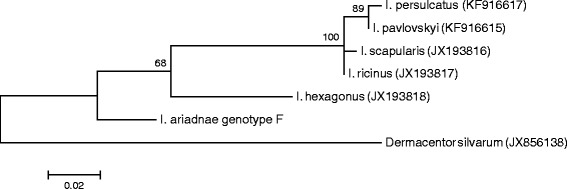
**Phylogenetic comparison of the subolesin sequence of** ***Ixodes ariadnae*** **from the present study with related sequences from the GenBank.** Branch lengths correlate to the number of substitutions inferred according to the scale shown.

The third target of molecular analyses in this study was the subolesin gene. It has an evolutionarily conserved function/sequence and plays a role (among the others) in immunity, development and reproduction of ixodid ticks [9]. Sequencing of this gene was only successful from *I. ariadnae* (KM455971). The subolesin gene of *I. ariadnae* clustered the closest to (and had the highest, 95% sequence homology with) that of *I. hexagonus* (Figure 9), whereas it had only 91% sequence homology with that of *I. ricinus*. In comparison with the latter species, the subolesin gene of *I. ariadnae* contained a 10 bp long and several smaller inserts. Thus, the translated protein had 86 to 88% similarity (and 16 or 7 amino acids longer strand) in comparison with that of *I. hexagonus* and *I. ricinus*, respectively.

These results also imply, that among all the hard tick species for which the subolesin gene and translated protein was reported so far (in [15]: 10 species of six genera were included), *I. ariadnae* has the longest known subolesin gene/protein (with 191 amino acids). Taking into account, that according to phylogenetical analyses subolesin sequences may have evolved from longer sequences in *Ixodes* spp. to shorter ones in other tick genera [15], it is possible that *I. ariadnae* is an ancient tick species. The importance of the latter finding, i.e. a new type of subolesin (protective) gene is further increased by the fact, that this gene is regarded as one of the most likely candidates among the targets of vaccines to control tick-infestations [9,15].

### Conclusions

These results support the taxonomic status of *I. ariadnae* as a separate species, and illustrate the morphological and phylogenetic differences between the three known European bat tick species. According to its subolesin gene *I. ariadnae* may have a long evolutionary history.
